# Evaluation of the Virulence of Low Pathogenic H9N2 Avian Influenza Virus Strains in Broiler Chickens

**DOI:** 10.3390/vetsci10120671

**Published:** 2023-11-24

**Authors:** Márta Bóna, József Földi, Lilla Dénes, Andrea Harnos, Bettina Paszerbovics, Míra Mándoki

**Affiliations:** 1 Department of Animal Hygiene, Herd Health and Mobile Clinic, University of Veterinary Medicine, István utca 2, 1078 Budapest, Hungary; 2 Euvet Bt., Asbóth utca 2, 2100 Gödöllő, Hungary; 3 National Laboratory of Infectious Animal Diseases, Antimicrobial Resistance, Veterinary Public Health and Food Chain Safety, University of Veterinary Medicine Budapest, István utca 2, 1078 Budapest, Hungary; denes.lilla@univet.hu; 4 Department of Biostatistics, University of Veterinary Medicine, István utca 2, 1078 Budapest, Hungary; harnos.andrea@univet.hu (A.H.); paszerbovics.bettina@univet.hu (B.P.); 5 Department of Pathology, University of Veterinary Medicine, István utca 2, 1078 Budapest, Hungary; mandoki.mira@univet.hu

**Keywords:** avian influenza, H9N2, virulence, broiler chicken, pathology, immunohistochemistry, PCR

## Abstract

**Simple Summary:**

The H9N2 subtype is widespread worldwide and is endemic among low-pathogenic avian influenza (LPAIV) viruses in the Middle East, North Africa and Asia. The virulence of H9N2 viruses of Middle Eastern and North African origin was investigated by different methods (necropsy, histopathology, immunohistochemistry and quantitative RT-PCR). Clear difference was demonstrated between H9N2 isolates belonging to the same lineage (G1): A/chicken/Morocco/2021/2016 strain proved to be significantly more virulent than those of the Egyptian and Saudi Arabian ones, which showed no remarkable difference.

**Abstract:**

Our study aimed to investigate the virulence of three recent H9N2 LPAIV strains belonging to the G1 lineage, isolated from field infections in North Africa and the Middle East. Three-week-old commercial broiler chickens (in total 62) were included and randomly allocated into three infected test groups and one control group. Each test group was inoculated intranasally/intratracheally with one of the three H9N2 isolates at a dose of 10^8^ EID_50_ virus. The control group received phosphate-buffered saline (PBS) via the same route of application. The pathogenicity was evaluated based on clinical signs and gross pathological and histopathological lesions, the viral antigen load was assessed through immunohistochemistry staining (IHC), and a semi-quantitative detection of the genetic material was conducted via a real-time PCR. Our findings confirmed the obvious respiratory tract tropism of the virus strains with variable renal tropism. In contrast to the highly pathogenic AIVs, the tested H9N2 strains did not show replication in the central nervous system. The virus presence and lesions, mainly in the respiratory tract, were predominant on dpi 5 and significantly reduced or disappeared by dpi 11. A clear difference was demonstrated among the three isolates: the A/chicken/Morocco/2021/2016 strain proved to be significantly more virulent than the Egyptian and Saudi Arabian ones, which showed no remarkable difference.

## 1. Introduction

The LPAI H9N2 subtype of avian influenza is currently the most prevalent strain worldwide. The successful dissemination of this subtype can be attributed to major migratory bird routes and live bird markets in specific regions [1]. 

The H9N2 avian influenza virus (AIV) was first isolated in 1966 from turkeys in the USA. In Eurasia, the earliest records describe the virus in clinically healthy domestic ducks in Hong Kong between 1975 and 1985. The genetic testing of early viruses suggests multiple introductions from wild waterfowl into duck flocks. A significant change occurred in the epidemiology of the virus during the 1990s. Between 1994 and 1997, H9N2 infection was detected in chickens in China’s Guangdong Province, and by the end of the 1990s, the virus adapted to chickens had spread to almost all provinces of China with the help of live animal markets [1,2]. The phylogenetic characteristics classified the virus into three distinct lineages named after their prototype strains, i.e., the G1 linage (A/Quail/Hong Kong/G1/1997); the BJ94 lineage, also known as Y280 (A/Chicken/Beijing/1/94); and the Y439 lineage, often mentioned as the ‘Korean’ lineage [3,4,5]. Further classification of the geographically most widespread G1 lineage is not fully clarified: some authors describe four sub-lineages designated as A, B, C, and D [5,6], while others distinguish ‘Western’ (W) and ‘Eastern’ (E) sub-lineages [4].

H9N2 strains isolated in the Middle East and North Africa are genetically related. El Khantour et al. highlighted the clear relationship between Moroccan viruses and G1 lineage viruses isolated in the Middle East [7,8]. In North Africa, infection with H9N2 LPAIV was first detected in Morocco in January 2016 [9]. The suspicion of disease was based on severe respiratory signs, and H9N2 virus infection was confirmed via RT-PCR. A few genetic and phylogenetic differences were observed between the isolates in 2016 and 2018, despite vaccination since July 2016 [8,10]. In late 2017, elevated poultry death rates were identified on Algerian farms where H9N2 avian influenza outbreaks had taken place. A subsequent examination of the viruses from the affected sites indicated that they were monophyletic of the G1 lineage [11,12]. A recent paper found that H9N2 strains isolated from vaccinated chicken farms in Egypt belonged to the G1-B sub-lineage [6]. H9N2 LPAIV was detected in poultry in sub-Saharan Africa in 2017 [13]. Several domestic poultry-adapted lineages of H9N2 LPAIV have circulated widely, causing significant economic losses in Kenya, where poultry production plays an important role in the economy and livelihoods of rural households [14].

The primary clinical symptoms resulting from H9N2 LPAIV infection are anorexia and reduced water intake, followed by depression, coughing, sneezing, and dyspnea. Affected birds can be observed gasping for air with their mouths open, necks extended, and heads raised. In addition, field cases commonly exhibited sinus enlargement and ocular discharge, leading to the contamination of the adjacent nostril. The birds most severely affected by the disease died suddenly due to asphyxia caused by necrotic debris clogging the airway at the tracheal bifurcation. In infected birds, the most common visible defects were the opacity of the thoracic and abdominal air sacs, accompanied by mild congestion of the trachea and lungs. The kidneys were enlarged [10,15,16]. In affected flocks, the mortality rates ranged from 10 to 65% within five to seven days of the emergence of clinical symptoms. Additionally, a significantly reduced weight gain was reported. Morbidity and mortality significantly increased in cases of co-infection with other microorganisms that cause respiratory diseases. A disease prevalence of 58% was reported in an epidemiological survey conducted in Morocco [10].

In addition, H9N2 LPAIV has caused repeated human infections in Asia since 1998, raising public concern about increasing pandemic potential [17,18]. H9N2 has demonstrated public health significance, as it could not only infect humans directly, but also donates partial or even whole cassettes of internal genes to generate novel human lethal reassortments such as H5N1, H7N9, H10N8, and H5N6 viruses [19].

Our study aimed to investigate the virulence of three recent H9N2 LPAIV strains in an experimental infection model in broiler chickens.

## 2. Materials and Methods

### 2.1. Experimental Design

Three-week-old commercial broiler chickens (Ross 308) tested negative for antibodies to AI H9 and were inoculated intranasally and intratracheally (IN-IT). A total number of 62 broilers were included in the study; the design is presented in Table 1. At 21 days of age, the birds were randomly allocated into three (3) infected and one control group, and they were placed in separate isolators under the same conditions (22 °C, RH 60%, 16 h lighting program), fed with commercial grower feed, and supplied with water ad libitum during the 11 days post-infection (dpi) observation period.

The chickens were inoculated with 10^8^ EID_50_ virus in a dose volume of 0.2 mL. All three virus strains belong to the G1 lineage, within the G1-like sub-lineage based on the genome sequencing. The virulence of the virus strains was evaluated based on clinical signs, gross pathological and histopathological lesions, viral antigen load based on immunohistochemistry (IHC), and the semi-quantitative detection of the genetic material via real-time PCR.

### 2.2. Clinical Signs and Mortality

Clinical signs were observed, and mortality was recorded daily during the observation period (d0–d11). Clinical signs were scored as follows: 0 = no signs; 1 = mild respiratory signs with no systemic signs (e.g., increased respiration rate, some nasal discharge, eye redness); 2 = moderate respiratory signs (e.g., markedly increased respiration rate or labored breath), no general sign, or mild depression; 3 = severe respiratory signs (e.g., marked labored breath, gasping) and obvious depression.

### 2.3. Sample Collection

Sample collection was performed on the 5th and 11th dpi, targeting the acute and chronic phase of the disease. Oronasal and cloacal swabs were taken before euthanasia on the given days. The number of birds submitted for examination per treatment group is given in Table 1. The animals were euthanized (through an intravenous injection of pentobarbital sodium (Release 300 mg/mL) solution), gross pathological observations were recorded, and tissue samples from the trachea, lung, kidney, spleen, pancreas, and brain were taken for histopathology, IHC, and PCR. A necropsy and sample collection of the chickens that died up to dpi 5 were performed in the same way as of the euthanized ones, except for one chicken from group ‘C’ that died on dpi 6, on which only a gross pathological examination was performed.

### 2.4. Histopathological Staining

At necropsy, the tissue samples described above were collected in 10% neutral buffered formalin, fixed for approximately 24 h, and then processed through the standard techniques of washing, dehydration, and embedding (Paraffin Biowax Blue, BioGnost Ltd., Zagreb, Croatia). Sections of 1.5 µm thickness were cut from the paraffin-embedded organs using a standard microtome (Shandon Finesse, Thermo Fisher Scientific Inc., Waltham, MA, USA) and stained with hematoxylin and eosin (H&E) according to the standard method [20]. The slides were examined using a Nikon Eclipse E200 light microscope with achromatic Leica objectives (Nikon Instruments Inc., Melville, NY, USA) equipped with a camera (3D Histech Ltd., Budapest, Hungary).

### 2.5. Immunohistochemical Staining

Indirect two-step immunoreactions of monoclonal primary and peroxidase-labelled anti-mouse secondary antibodies were used with AEC (3-amino-9-ethylcarbazole) as the chromogen. The staining method and scoring system have been described in detail elsewhere [21]. The LPAI virus antigen appeared as a red precipitate in the nuclei of affected cells but may also have been observed in the cytoplasm.

### 2.6. Real-Time PCR

Organ samples were homogenized in 1 mL of sterile PBS using the Tissue Lyser II (Qiagen, Hilden, Germany) with 5 mm steel balls. The sample material was eluted from the swab heads in 1 mL of sterile PBS using the Tissue Lyser II (Qiagen). RNA was extracted with the cador Pathogen 96 QIAmp HT Liquid kit (Qiagen) using the QIAcube HT device, according to the manufacturer’s instructions. A four-microliter RNA extract was used as the template for the one-step RT-PCR (AIV qPCR TaqMan Fast Virus 1-Step Master Mix; Thermo Fisher Scientific Inc., Waltham, MA, USA). The primers and probe used for the amplification and detection of a fragment of the matrix gene were described previously [22].

The cutoff value of the method was set to Ct 36; i.e., samples with Ct < 36 were considered as positive and ≥36 as negative. The negative samples with no amplification were set to the maximum cycle number of 40.

### 2.7. Statistical Analysis

Reflecting the grade of severity of the clinical signs, visible lesions, and histopathological alterations, we applied a scoring system of 0–3, i.e., ‘no’, ‘mild’, ‘moderate’, and ‘severe’, for most of the observed parameters according to the recommendations [23]. Some observations, i.e., epithelial degeneration in the trachea and glomerulonephritis, were defined as ‘absent’ or ‘present’ (0–1). When a multiple organ (four air sacs) was scored, or different parameters were scored for the same organ (lung or kidney), the sum of scores was used for statistical evaluation. The IHC was evaluated as ‘no’, ‘low’, and ‘high’ virus antigen load (i.e., a scale of 0–2) for each tissue tested, as described in our previous paper [21].

As the clinical scores were collected on an ordinal scale, and since repeated measurements were taken on the same chicken, we employed a mixed-effects continuation ratio (CR) model in the three groups [24,25]. We included two fixed effects (group and time) and one random effect (the chicken ID).

We treated the PCR results as binary data, classifying Ct values less than 36 as positive and values between 36 and 40 as negative. Since, in some cases, positive results were rare, we employed penalized maximum likelihood logistic regression, providing predicted probabilities of observing a positive PCR result and allowing us to assess the dpi and group impact on this probability [26]. The model was fitted with the logistf function of the logistf package using the software R, version 4.2.1 (23 June 2022 ucrt).

For outcomes with more than two categories, like different levels of severity, we employed the cumulative link model (CLM). The CLM models the probability of falling into a specific category or lower compared to higher categories [27,28]. As a result of the CLM, we obtain odds ratios quantifying the change in odds for a change between categories in the predictors. In most cases, the predictors were the dpi and group; any deviations from this are outlined in Section 3. We conducted pairwise comparisons with the emmeans function of the emmeans package and the pairs function of the graphics package. This statistical method allows us to identify which groups we can find statistically significant differences between. For *p*-value adjustment, the Tukey method was applied. The cumulative link model was fitted with the clm function of the ordinal package using the software R, version 4.2.1 (23 June 2022 ucrt).

The mortality rate between groups was compared using Fisher’s exact test for the 3 × 2 contingency table. The correlation between the histopathology, IHC, and PCR results was tested using Spearman rank correlation. A statistical analysis was performed only if there was at least one value different from zero or different between challenged groups.

All observed parameters were considered physiological (i.e., no lesions) in the control group; hence, they were not included in the statistical analysis. The level of significance was set to *p* < 0.05.

## 3. Results

Neither clinical symptoms were observed, nor were gross pathological or histopathological lesions demonstrated; the IHC and PCR tests failed to detect any trace of the LPAIV infection in the control group during the study. Therefore, the results below are summarized for the three challenged groups (A, B, and C strain) only.

### 3.1. Clinical Signs and Mortality

Generally, respiratory signs, i.e., sniffling, gurgling, sneezing, wheezing, and, occasionally, dyspnea, in different grades of severity were observed in most of the challenged birds between 3 and 11 dpi, regardless of the challenge virus strain.

A significant difference was observed between strain A and C (*p* = 0.011) as well as between strain B and C (*p* = 0.0055); however, the statistical analysis revealed no significant difference between strain A and strain B (*p* = 0.8078)

**Figure 1:**
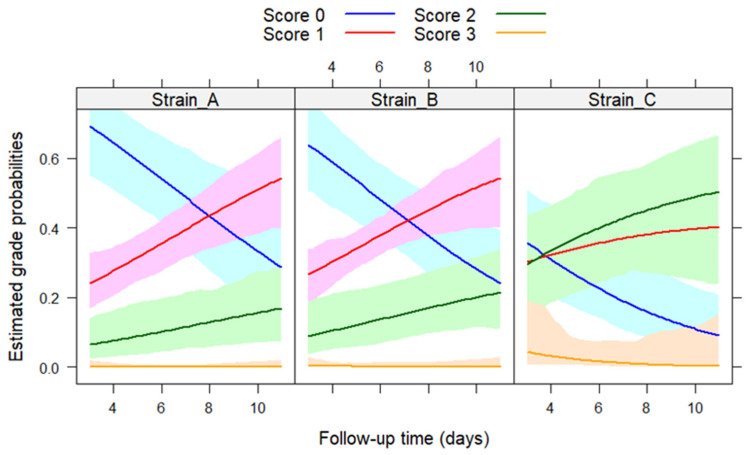
Predicted probabilities and their 95% confidence bands for each score category in the three groups during the follow-up period.

Figure 1 presents the effect plots illustrating how the probability of being in a specific grade category changed over the follow-up period in the three strains. Notably, in the case of strains A and B, similar patterns were observed, while for strain C, the chickens tended to be in higher-grade categories, indicating worse clinical conditions.

One chicken from group ‘A’ (5.5%) and four birds from group ‘C’ (22%) died at 4–6 dpi, while no mortality occurred in group ‘B’ (0%). The mortality rates showed no significant difference (Fisher’s exact test *p* = 0.113).

### 3.2. Gross Pathology

**Figure 2:**
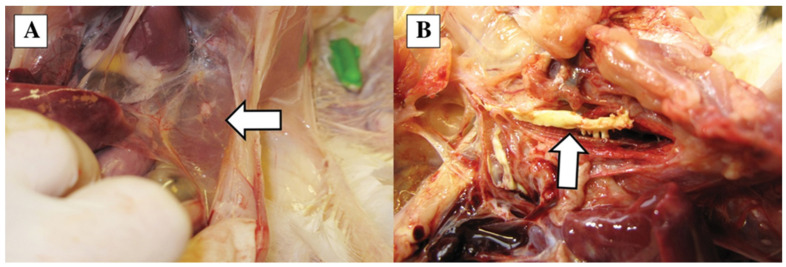
Post-mortem lesions: mild hyperemia in the air sac (arrow) (**A**) and fibrinous exudate (arrow) in the bifurcation of the trachea (**B**).

Macroscopic lesions were seen in the respiratory system, including ruddy or purple–red tracheal mucosa and hyperemia in the air sacs and congestion in the lungs (Figure 2A), occasionally with a caseous fibrin plug at the bifurcation (Figure 2B).

The number of affected chickens and the severity of the lesions are summarized in Table 2.

In the case of the trachea lesion scores, the dpi variable appeared to be significant (odds ratio (dpi 11/dpi 5): 3.7, *p* = 0.0158), which means that the odds of experiencing a reduction in lesion severity at dpi 11 are 3.7 times higher than at dpi 5. However, we could not detect significant differences between the three strains. In the analysis for air sacs, we could detect significant differences between strain A and B (odds ratio (A/B): 10.34, *p* = 0.0199) and strain A and C (odds ratio (A/C): 33.97, *p* = 0.0003)

### 3.3. Histopathology

Histopathological lesions were observed in the trachea, lungs, and kidneys, while no alteration was found in the spleen, pancreas, and brain of any chicken.

**Figure 3:**
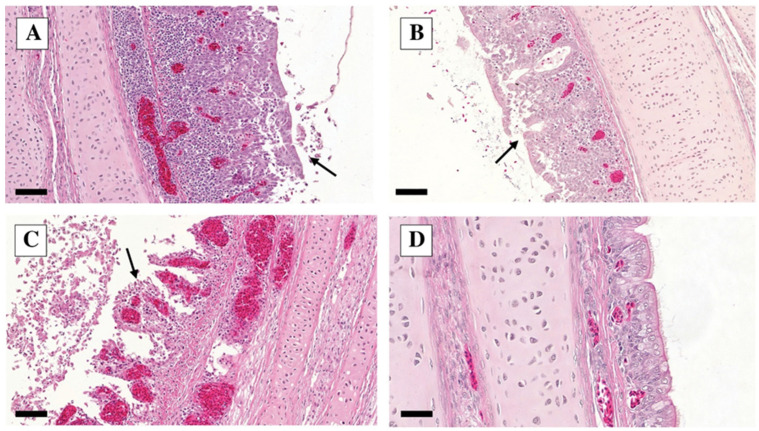
Trachea H&E staining on dpi 5. (**A**–**C**): challenge groups A–C—lymphocytic inflammation with epithelial degeneration (see arrows) 20×, bar = 100 µm. (**D**): control group—intact ciliated epithelial cells, 40×, bar = 50 µm.

The tracheal lesions were characterized by lymphocytic inflammation; i.e., inflammatory cells, mainly lymphocytes, infiltrated the epithelium with exfoliated epithelial cells and epithelial degeneration (Figure 3).

The former was evaluated on a 0–3 scale (see above); the latter was present in all birds examined on dpi 5 and absent in all birds on dpi 11, as demonstrated in Table 3, where the severity of the lymphocytic inflammation is also summarized.

In the case of the lymphocytic inflammation scores, the dpi variable appeared to be significant (*p* < 0.0001), which means that the severity of the lesions significantly decreased on dpi 11 compared to dpi 5 (OR: 262), and we also could detect significant differences between strains A and C (OR: 8.36, *p* = 0.0116).

The lung lesions comprised interstitial pneumonia (i.e., focal infiltration of the perivascular and peribronchial tissue by lymphoid cells and macrophages), and catarrhal bronchitis, (i.e., inflammatory exudate, infiltration, and edema). The number of affected birds with different grades of severity is given in Table 4.

We calculated the sum of the scores of interstitial pneumonia, bronchitis, catarrhal infiltration, and edema. The analysis results show that the severity of the lesions significantly decreased on dpi 11 compared to dpi 5 (OR: 281, *p* < 0.0001), but between the three strains, we could not detect a significant difference.

**Figure 4:**
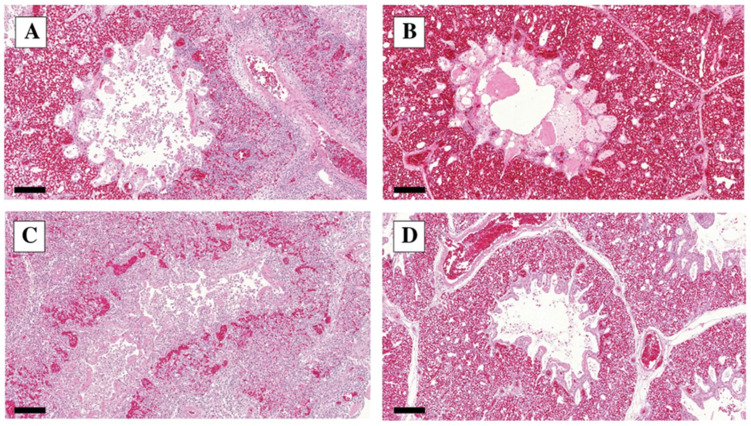
Lung H&E staining dpi 5. (**A**) Lung infected with A strain: interstitial inflammation score 3, bronchitis score 3, catarrhal infiltration score 2, edema score 2; in the alveolus, many exfoliating alveolar epithelial cells are seen. H&E, 10×, bar = 200 µm. (**B**) Lung infected with B strain: interstitial inflammation score 2, bronchitis score 2, catarrhal infiltration score 2, edema score 1. H&E, 10×, bar = 200 µm. (**C**) Lung infected with C strain: interstitial inflammation score 3, bronchitis score 3, catarrhal infiltration score 3, edema score 2. H&E, 10×, bar = 200 µm. (**D**) Control lung: no interstitial inflammation, bronchitis, catarrhal infiltration, or edema. H&E, 10×, bar = 200 µm.

The characteristic lung lesions found via histopathology are presented in Figure 4.

Histopathological lesions in the kidneys were observed only in chickens examined on dpi 5, as shown in Table 5, where the types of lesions, severity, and the numbers of affected chickens are also presented.

**Figure 5:**
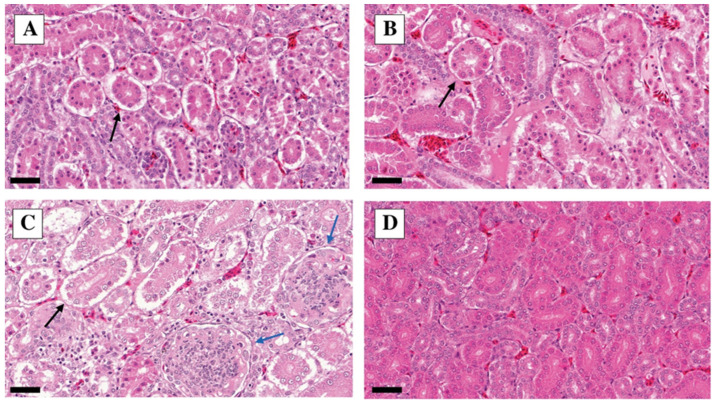
Kidney H&E staining dpi 5; 40×, bar = 50 µm. (**A**) Kidney infected with A strain: diffuse, moderate tubulonephrosis score 2; the tubular cells detach from the basal membrane (black arrow); karyorrhexis, karyopyknosis. (**B**) Kidney infected with B strain: focal tubulonephrosis score 2. (**C**) Kidney infected with C strain: diffuse lymphocytic inflammation in the interstitium score 1, tubulonephrosis (tubular cells detach from the basal membrane (black arrow)), karyopyknosis, karyorrhexis score 3, glomerulonephritis: mesangium proliferation (blue arrow). (**D**) Control kidney: intact tubule cells on the basal membrane.

The lesions were characterized mainly by tubulonephrosis (i.e., regressive lesions of the tubular epithelial cells) and glomerulonephritis (lympho-histiocytic infiltration), with mild lymphocytic inflammation in a few cases observed only on dpi 5. The lymphocytic inflammation, tubulonephrosis, and glomerulonephritis scores were modelled based on group variables, and the only significant difference was found in the case of tubulonephrosis between strain A and C (OR: 31.95, *p* = 0.0026), and strain B and C (OR: 248.25, *p* = 0.0002). The histopathological lesions of the kidney are shown in Figure 5.

### 3.4. IHC

**Figure 6:**
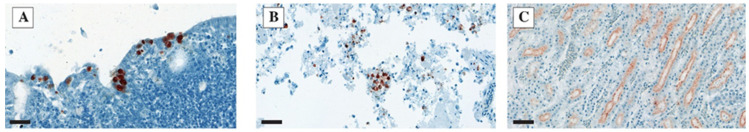
Immunohistochemical staining dpi 5. (**A**) Visible dark red deposits in the nuclei of the exfoliating epithelial cells in the trachea. H&E counterstain, 40×, bar = 50 µm. (**B**) Dark red virus antigen in the exfoliating alveolar epithelial cells in the lung. H&E counterstain, 40×, bar = 50 µm. (**C**) Dark red deposits in the cytoplasm of the renal tubular epithelium in the kidney. H&E counterstain, 40×, bar = 50 µm.

Viral antigens were detected in approximately 50% of the challenged birds in the trachea, lung, and kidney samples taken on dpi 5. Positive results appeared occasionally (two cases) in the spleen, but no virus antigen was demonstrated in the pancreas and brain. All samples tested negative on dpi 11, as shown in Table 6, where the IHC results are summarized. Positive IHC staining appeared as dark red deposits in the nuclei of exfoliating epithelial cells in the trachea, alveolar epithelial cells in the lung, and more so in the cytoplasm of the renal tubular epithelium (Figure 6). Whole-cell staining was more common in the kidney, whereas the clumping of nuclei was seen in the respiratory tract.

The tracheal and kidney IHC scores were modeled based only on the group variable. In the analysis of the tracheal IHC scores, no significant differences were observed among the three strains. However, in the context of the kidney IHC scores, a significant difference between strain A and strain C (OR: 11.37, *p* = 0.0184) was found.

### 3.5. PCR

**Figure 7:**
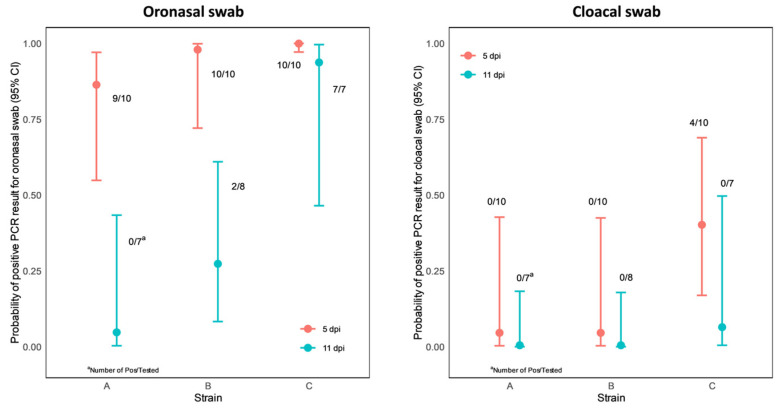
Representation of probability point estimates enclosed in 95% confidence intervals for oronasal (**left**) and cloacal (**right**) swab PCR results by strain.

The virus excretion was evaluated via PCR from oronasal and cloacal swabs taken on dpi 5 and 11. All but one (group ‘A’) chicken shed the challenge virus via the oronasal swab on dpi 5; however, it was markedly decreased on dpi 11, with a high difference between the groups. Figure 7 shows the estimated effects of the strain and dpi on the probability of obtaining positive PCR results. In the case of the oronasal swab PCR results, the dpi variable appeared to be significant (*p* < 0.0001), and a significant difference was obtained between strains A and C (*p* = 0.0175). In the case of the cloacal swab, the dpi variable was not significant and we were not able to detect significant differences between the strains.

The challenge strains could be detected via PCR in the respiratory tract at different levels on dpi 5 and 11 in the following:

- In the trachea: all birds but one on dpi 5 and in the majority of them (18 out of 22, 82%) on dpi 11. This observation is supported by the model that neither the dpi nor the strain has a significant effect on the positive PCR result;
- In the air sacs: 80% (24 out of 30) of the chickens were found positive on dpi 5 and 9% (2 out of 22) on dpi 11. Accordingly, the dpi variable appeared to be significant (*p* < 0.0001), but no significant difference was obtained between the three strains;
- In the lungs: 90% (27 out of 30) of the chickens were found positive on dpi 5 and 32% (7 out of 22) on dpi 11. In the case of the lung PCR results, the dpi variable appeared to be significant (*p* < 0.0001), and a significant difference was obtained between strains A and C (*p* = 0.0093) and strains B and C (*p* = 0.0257).

**Figure 8:**
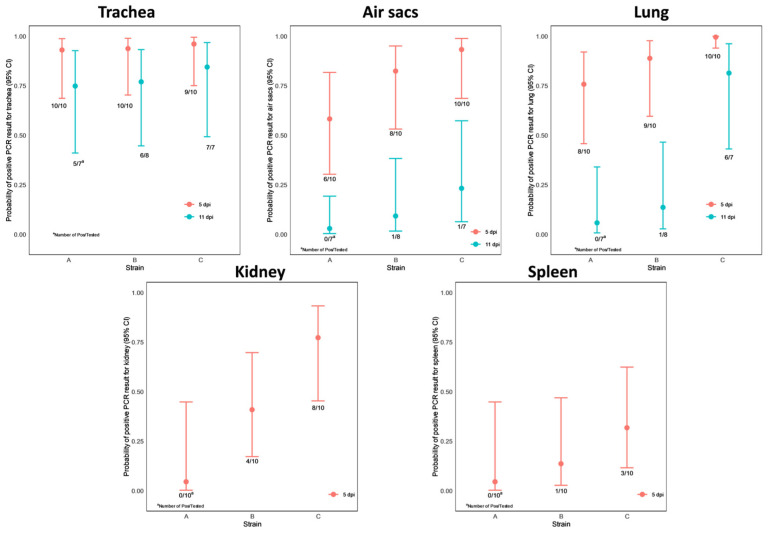
Representation of probability point estimates enclosed in 95% confidence intervals for trachea, air sacs, lung, kidney, and spleen PCR results by strain.

Figure 8 shows the estimated effects of the strain and dpi on the probability of obtaining positive PCR results in the trachea, air sacs, lung, kidney, and spleen.

The virus genome was occasionally detected in the kidney and spleen on dpi 5 only. In the spleen, no significant differences were observed among the three strains, while a significant difference was detected between strains A and C (*p* = 0.0351) in the case of the kidney PCR results.

### 3.6. Correlation between the Diagnostic Methods

Spearman’s rank correlation coefficients (rs) were calculated to compare the similarity of the scores obtained via histopathology versus IHC and histopathology versus PCR in the trachea, lung, and kidney, since the respiratory tract and kidney proved to be the tissues where the challenge viruses caused different severities of remarkable lesions. A statistically significant (*p* < 0.0500) but moderate positive correlation was demonstrated between the histopathology and IHC results (rs = 0.37 for trachea and lung; 0.47 for kidney) and similarly, a significant and moderate, however, negative correlation was proven between the histopathology scores and Ct values via PCR (rs = [−0.39] for kidney, [−0.48] for lung and [−0.6] for trachea).

## 4. Discussion

The experimental infection of commercial broiler chickens with H9N2 LPAIV strains usually resulted in mild to moderate clinical signs, mostly appearing on the head and upper respiratory tract, like lacrimation, eye and/or facial swelling, sneezing, gasping, and nasal discharge. Mild depression and reduced feed and water intake were also often observed. These symptoms appeared on dpi 2–3 and persisted until dpi 5–7; the birds usually recovered by dpi 10–12 with no mortality [29,30]. Exceptionally, more severe clinical signs—i.e., general depression, cowering, huddling, ruffled feathers, and mild respiratory signs including coughing, sneezing, and sometimes wheezing, especially in mycoplasma-positive subgroups, with a mortality rate of up to 40%—were reported following experimental infection with a H9N2 strain isolated from the kidney [31]. Hyperemia, petechial hemorrhages, occasional focal fibrin deposition in the trachea (at the bifurcation) and lungs, and thickened opaque air sacs were observed by others as gross pathological lesions, occasionally including swollen kidneys [15,30,31,32].

In our study, generally, mild or moderate respiratory signs (i.e., increased respiration rate, some ocular/nasal discharge, eye redness) with no systemic signs were observed between dpi 3 and 11. Severe respiratory signs with obvious depression were seen occasionally, only on dpi 4–5 and in the group inoculated with the A/chicken/Morocco/2021/2016 H9N2 virus (group ‘C’). The probability of achieving clinical scores ≥ 1 was significantly higher in group ‘C’ than in groups ‘A’ and ‘B’ (inoculated with A/chicken/Saudi Arabia/8616/2016 H9N2 or A/chicken/Egypt/4531/2016 H9N2, respectively). Mortality occurred mostly on dpi 5 (three chickens from group ‘C’), with one case on dpi 4 (group ‘A’) and one on dpi 6 (group ‘C’). Although four deaths out of 18 in group ‘C’ seems higher compared to the one in group ‘A’ and 0 in group ‘B’, the difference is not significant, probably due to the relatively low sample size. These findings are in line with those described by others for experimental infections with H9N2 viruses. Nevertheless, it is noteworthy that field reports usually mention more severe clinical symptoms and higher mortality rates than in experimental infection studies, which may well be explained by the numerous predisposing factors and co-infections with respiratory microorganisms in the field. Visible pathological lesions were limited to the (upper) respiratory tract, mainly characterized by tracheitis and airsacculitis, occasionally with caseous fibrin plugs at the bifurcation. The tracheal lesions were significantly more severe on dpi 5 than on dpi 11, with no significant difference between groups, while the air sacs showed significantly more severe lesions in group ‘C’ than those of groups ‘A’ and ‘B’ on dpi 5.

Similarly, for the gross lesions, the histopathological changes were predominant in the respiratory tract, characterized by inflammatory and necrotic processes, as reported by all experimental infection studies. The proliferation of goblet cells was seen first in the trachea, followed by the sloughing of the tracheal epithelium and deciliation with the lymphocytic infiltration of the mucosa. The lungs showed congestion and perivascular hemorrhages, with heterophil cell infiltration and the collapse of the alveoli with edema around the blood vessels. Pale bluish mucus and heterophil accumulation in the lumen of secondary bronchioles were also present [15,29,30,31].

The kidneys showed interstitial congestion, hemorrhage, vacuolar degeneration in the tubular epithelium, glomerular degeneration, and collapse with periglomerular fibrosis. The frequency of kidney lesions was quite variable between studies, most probably depending on the tissue tropism of the H9N2 strains used for experimental infection [29,30,31]. Occasionally, hepatocytic swelling and necrosis were also observed in the liver [29], and the gastrointestinal tract was affected [32].

In our study, histopathological lesions in the trachea were characterized by lymphocytic inflammation with exfoliating epithelial cells and epithelial degeneration. Lymphocytic inflammation was evident at both dpi 5 and 11, and it was significantly more severe in group ‘C’ than in group ‘A’ on dpi 5; however, it was not confirmed by the IHC and RT-PCR results of the trachea samples. The epithelial degeneration had resolved until dpi 11. The histopathological lesions in the lungs were characterized by interstitial pneumonia, bronchitis, catarrhal infiltration, and edema, with no significant difference between groups; however, a significant decrease in severity between dpi 5 and 11 in each group was observed. Histopathological examinations revealed mainly tubulonephrosis and glomerulonephritis in the kidney, with occasional lymphocytic inflammation. Tubulonephrosis occurred with significantly higher frequency in groups inoculated with the A/chicken/Morocco/2021/2016 H9N2 virus than those inoculated with the Egyptian and Saudi Arabian strains.

Positive immunolabeling for H9N2 viral antigens (i.e.,: virus load) was demonstrated as dark brown granules within the desquamated tracheal epithelium [15] in the nuclei of pulmonary epithelial cells, as well as in the nuclei or cytoplasm of necrotic renal tubular epithelium by ABP (avidin–biotin–peroxidase) in formalin-fixed specimens [30].

The virus load was demonstrated via IHC in our study in the trachea, lung, kidney, and a few cases of spleen sections on dpi 5 only, with no significant difference between groups except for the kidney, where the fitted cumulative link model showed significantly higher odds for the presence of the viral antigen in the group infected with the Moroccan strain.

Virus excretion is usually tested via PCR of oronasal/oropharyngeal and cloacal swabs. Likewise, for the Bangladesh H9N2 strain [32], we also found a remarkable virus shedding via the oronasal route, and much less of the virus was excreted via the feces and urine. Significantly more of the virus was shed via an oronasal route on dpi 5 than dpi 11 in all groups, and the same statistical result was obtained on dpi 5 comparing groups ‘C’ and ‘A’. Interestingly, virus shedding seemed to be prolonged in local breed (Sonali) chickens than in commercial broiler hybrids [32].

The virus genome was detected in the trachea, air sacs, and lungs, both on dpi 5 and 11; however, a significant decrease in the copy number was demonstrated from dpi 5 to 11. The kidney and spleen samples showed positive PCR results only on dpi 5. Significantly more virus genes were detected in the kidneys in group ‘C’ than those of group ‘A’.

No H9N2 viruses were detected with any laboratory diagnostic method in the pancreas and brain of the inoculated birds.

We tried to determine the possibly existing difference in virulence between H9N2 LPAIV strains of different origins based on several parameters. These wider diagnostic tools allowed us to investigate the correlation between histopathology vs. IHC and histopathology vs. PCR. A significant but moderate correlation, without controversial results, was demonstrated.

The A/chicken/Morocco/2021/2016 H9N2 virus proved to be more virulent in our study compared to the Egyptian- and Saudi Arabian-origin strains. However, further investigations, including more H9N2 LPAIVs from different geographical origins and increasing the frequency of sampling, may help to establish the difference in virulence between the strains more precisely.

## 5. Conclusions

Our findings confirmed the obvious respiratory tract tropism with variable renal tropism of the H9N2 LPAIV strains studied. Our AIV strains showed no replication in the central nervous system at all. The presence of the viruses and the lesions caused by them were predominant on dpi 5 and significantly reduced or disappeared on dpi 11. A clear difference was demonstrated between the tested H9N2 isolates that belonged to the same subtype (H9N2) and same lineage (G1): the A/chicken/Morocco/2021/2016 strain proved to be significantly more virulent than the Egyptian and Saudi Arabian strains, which showed no remarkable diversion. Further studies are needed to investigate the genetic background of these viruses to determine the possible genetic determinants for the increased virulence of an H9N2 virus strain.

## Figures and Tables

**Table 1:** Study design: group codes, H9N2 strains used for IN-IT inoculation, and the number of chickens examined.

Group *	Challenge Strain	No. of Chickens Examined	
		dpi 5 **	dpi 11
A	A/chicken/Saudi Arabia/8616/2016 H9N2	11(1) ***	7
B	A/chicken/Egypt/4531/2016 H9N2	10	8
C	A/chicken/Morocco/2021/2016 H9N2	10(4) ***	7
Control	PBS buffer	3	6

* Study group codes, ** dpi: day post-infection. *** numbers in brackets indicate the mortality due to the disease.

**Table 2:** Gross pathological lesions were observed in the respiratory tract.

H9N2 Strain	‘A’	‘B’	‘C’	‘A’	‘B’	‘C’
	dpi 5			dpi 11		
Trachea						
No *	2 **	6	1	4	1	7
Mild	1	1	1	1	0	0
Moderate	7	1	3	2	7	0
Severe	1	2	6	0	0	0
Air sacs						
No	0	1	0	5	1	0
Mild	11	5	3	2	3	4
Moderate	0	4	6	0	4	1
Severe	0	0	2	0	0	2

* Severity of the lesions see also Section 2.7, ** Number of affected chickens.

**Table 3:** Histopathological lesions in the trachea.

H9N2 Strain	‘A’	‘B’	‘C’	‘A’	‘B’	‘C’
	dpi 5			dpi 11		
Lymphocytic inflammation						
No *	0 **	0	0	7	1	3
Mild	0	1	0	0	3	3
Moderate	7	5	1	0	4	1
Severe	4	4	9	0	0	0
Epithelial degeneration						
No	0	0	0	7	8	7
Yes	11	10	10	0	0	0

* Severity of the lesions see also Section 2.7; ** Number of affected chickens.

**Table 4:** Histopathological lesions in the lungs.

H9N2 Strain	‘A’	‘B’	‘C’	‘A’	‘B’	‘C’
	dpi 5			dpi 11		
Interstitial pneumonia						
No *	1 **	2	1	7	8	7
Mild	3	2	4	0	0	0
Moderate	5	3	2	0	0	0
Severe	2	3	3	0	0	0
Bronchitis						
No	1	0	0	4	6	3
Mild	0	0	1	3	1	3
Moderate	5	7	5	0	1	1
Severe	5	3	4	0	0	0
Catarrhal infiltration						
No	1	2	1	6	7	6
Mild	5	4	1	0	0	0
Moderate	5	2	4	0	1	1
Severe	0	2	4	1	0	0
Edema						
No	0	1	0	7	8	7
Mild	1	4	0	0	0	0
Moderate	8	5	5	0	0	0
Severe	2	0	5	0	0	0

* Severity of the lesions see also Section 2.7; ** Number of affected chickens.

**Table 5:** Histopathological lesions in the kidneys.

H9N2 Strain	‘A’	‘B’	‘C’	‘A’	‘B’	‘C’
	dpi 5			dpi 11		
Lymphocytic inflammation						
No *	9 **	8	8	7	8	7
Mild	2	1	2	0	0	0
Moderate	0	1	0	0	0	0
Severe	0	0	0	0	0	0
Tubulonephrosis						
No	1	3	0	7	8	7
Mild	3	5	0	0	0	0
Moderate	7	2	3	0	0	0
Severe	0	0	7	0	0	0
Glomerulonephritis						
No	3	5	0	7	8	7
Yes	7	5	10	0	0	0

* Severity of the lesions see also Section 2.7; ** Number of affected chickens.

**Table 6:** Viral antigen detection via IHC in different tissues.

H9N2 Strain	‘A’	‘B’	‘C’	‘A’	‘B’	‘C’
	dpi 5			dpi 11		
Trachea						
No *	5 **	7	4	7	8	7
Low	6	2	2	0	0	0
High	0	1	4	0	0	0
Lung						
No	7	10	3	7	8	7
Low	3	0	5	0	0	0
High	1	0	2	0	0	0
Kidney						
No	9	6	1	7	8	7
Low	2	4	4	0	0	0
High	0	0	5	0	0	0
Spleen						
No	10	10	9	7	8	7
Low	1	0	1	0	0	0
High	0	0	0	0	0	0

* Severity of the lesions see also Section 2.7; ** Number of affected chickens.

## Data Availability

Not applicable.
